# Potential Predictive and Prognostic Value of Biomarkers Related to Immune Checkpoint Inhibitor Therapy of Triple-Negative Breast Cancer

**DOI:** 10.3389/fonc.2022.779786

**Published:** 2022-04-29

**Authors:** Qiaorui Tan, Sha Yin, Dongdong Zhou, Yajing Chi, Xiaochu Man, Huihui Li

**Affiliations:** Department of Breast Medical Oncology, Shandong Cancer Hospital and Institute, Shandong First Medical University and Shandong Academy of Medical Sciences, Jinan, China

**Keywords:** predictive biomarkers, immunotherapy, triple-negative breast cancer, immune checkpoint inhibitors, prognostic biomarker

## Abstract

As an aggressive subtype of breast cancer, triple-negative breast cancer (TNBC) is associated with poor prognosis and lack of effective therapy, except chemotherapy. In recent years, immunotherapy based on immune checkpoint (IC) inhibition has emerged as a promising therapeutic strategy in TNBC. TNBC has more tumor-infiltrating lymphocytes (TILs) and higher rate of mutation and programmed cell death ligand-1 (PD-L1) expression than other subtypes of breast cancer have. However, previous studies have shown that monotherapy has little efficacy and only some TNBC patients can benefit from immunotherapy. Therefore, it is important to identify biomarkers that can predict the efficacy of IC inhibitors (ICIs) in TNBC. Recently, various biomarkers have been extensively explored, such as PD-L1, TILs and tumor mutational burden (TMB). Clinical trials have shown that PD-L1-positive patients with advanced TNBC benefit from ICIs plus chemotherapy. However, in patients with early TNBC receiving neoadjuvant therapy, PD-L1 cannot predict the efficacy of ICIs. These inconsistent conclusions suggest that PD-L1 is the best to date but an imperfect predictive biomarker for efficacy of ICIs. Other studies have shown that advanced TNBC patients with TMB ≥10 mutations/Mb can achieve clinical benefits from pembrolizumab. TILs also have potential predictive value in TNBC. Here, we select some biomarkers related to ICIs and discuss their potential predictive and prognostic value in TNBC. We hope these biomarkers could help to identify suitable patients and realize precision immunotherapy.

## 1 Introduction

Among women, breast cancer (BC) is the malignant tumor with the highest morbidity and the second highest mortality (1–4). BC can be divided into four subtypes on the basis of expression of estrogen receptor (ER), progesterone receptor (PR), human epidermal growth factor receptor 2 (HER2), and Ki-67 as follows: luminal A, luminal B, HER2-enriched, and triple-negative (TN). TNBC accounts for 12%–17% of BC, and compared with other subtypes, has specific characteristics including earlier onset age, higher metastatic potential, and worse prognosis (5, 6). As a heterogeneous disease, TNBC can be classified into multiple subtypes by different detection methods. According to transcriptome data from the Chinese population, TNBC can be divided into four subtypes: immunomodulatory (IM), luminal androgen receptor, mesenchymal-like, and basal-like and immune suppressed (7, 8). Because TNBC lacks expression of ER and PR and has little or no HER2 expression, it has become the most refractory BC, and chemotherapy is still the most important treatment regimen. Once the tumor has progressed, TNBC is often incurable and the overall survival (OS) is only 10–13 months (9, 10). Therefore, to extend the survival of TNBC patients, a novel treatment strategy is urgently needed.

Recently, immunotherapy has been the focus of investigation in tumor therapy. At present, immunotherapy has shown strong activity against some tumor types such as melanoma and non-small cell lung cancer (NSCLC). Tumor immunotherapy, because of its reliable efficacy and tolerable safety, is regarded as the most promising treatment after surgery, chemotherapy, radiotherapy and targeted therapy (11). The most frequently used immunotherapeutic drugs are immune checkpoint inhibitors (ICIs), such as programmed cell death protein 1 (PD-1)/PD ligand 1 (PD-L1) inhibitors and anti-cytotoxic T lymphocyte antigen-4 (CTLA-4) agents. ICIs can increase lymphocytic cytotoxicity and proliferation by interrupting the binding of IC receptors and ligands to exert antitumor effects. Compared with other subtypes of BC, TNBC has higher frequency of copy number changes, genetic instability, and structural rearrangements, which contribute to its high mutation rate (7, 12). The high mutation rate in TNBC is associated with high lymphocyte infiltration and increased PD-L1 expression (13–15). Both immune cells and immunostimulators are enriched in the IM subtype of TNBC (7). These indicate that patients with TNBC, especially IM subtype, may benefit from ICIs. Therefore, increasing numbers of clinical trials have investigated the efficacy of ICIs for treatment of TNBC, and have shown promising results (16, 17). In the IMpassion 130 study, patients with metastatic or locally advanced unresectable TNBC treated with atezolizumab plus nab-paclitaxel had longer progression-free survival (PFS) in the intention-to-treat population and PD-L1-positive subgroup compared with patients treated with placebo plus nab-paclitaxel. Clinically meaningful prolonged OS was observed in the PD-L1-positive metastatic TNBC (mTNBC) subgroup treated with atezolizumab plus nab-paclitaxel (17, 18). Similar results were seen in the KEYNOTE-355 study, indicating that chemotherapy plus pembrolizumab significantly improved PFS compared with chemotherapy alone for PD-L1-positive patients with metastatic or locally advanced unresectable TNBC (19). Based on these results, pembrolizumab plus chemotherapy are strongly recommended by version 1.2021 of the National Comprehensive Cancer Network (NCCN) guidelines for BC as a first-line regimen in patients with locally advanced or mTNBC with PD-L1 expression.

However, not all TNBC patients can benefit from ICIs. The KEYNOTE-119 study showed that pembrolizumab did not prolong OS significantly in previously treated mTNBC patients, compared with chemotherapy (20). Similarly, in the IMpassion 131 study, paclitaxel combined with atezolizumab did not significantly prolong PFS or OS in the intention-to-treat population (21). These results question the efficacy of ICIs in TNBC. Therefore, it is necessary to identify biomarkers for the efficacy of ICIs to help select patients who could benefit from immunotherapy, and to guide the rational application of such drugs in clinical practice. Besides, some of these biomarkers also have potential prognostic value in TNBC. This review aims to summarize the recent development of the most-discussed biomarkers, which might help to predict the efficacy of immunotherapy and prognosis in TNBC patients.

## 2 Potential Predictive and Prognostic Value of the Biomarkers Related to ICIs in TNBC

**Figure 1:**
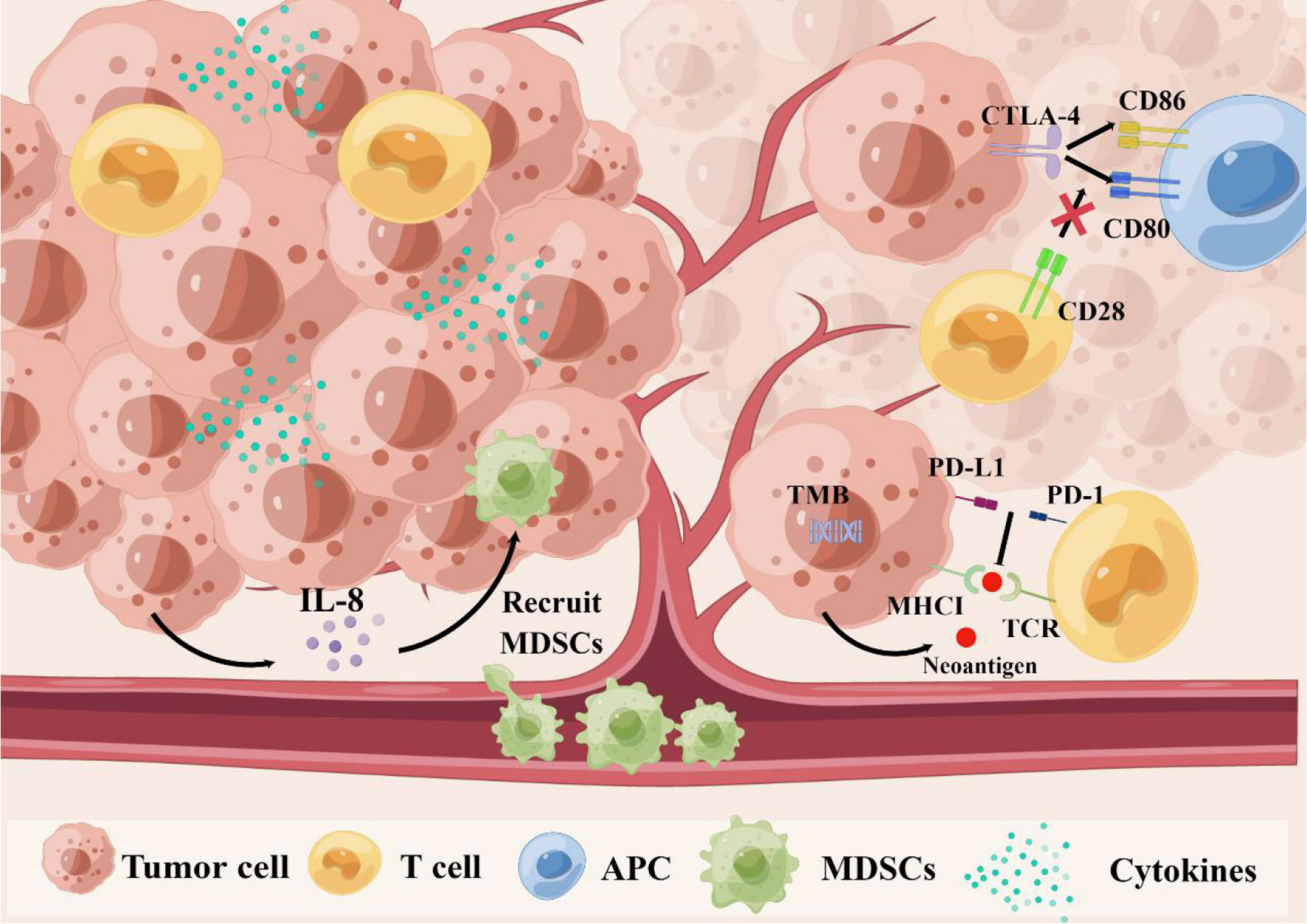
The relationship between biomarkers and immune resistance. First, TMB might lead to new antigens and enhance immunogenicity. Second, the PD-1 combined with PD-L1 can transmit inhibitory signals and reduce immune activation, which leads to the immune escape of tumor cells. Third, CTLA-4 can compete with CD28 to bind to CD80 and CD86 on antigen-presenting cells (APC), and inhibit the activation signal. Fourthly, cytokines can regulate proliferation, differentiation and function of immune cells, tumor microenvironment, and even affect migration of cancer cells. Especially, tumor cells secrete IL-8 to recruit MDSCs into the tumor microenvironment to induce immunosuppression, and promote tumor progression. CTLA-4, cytotoxic T lymphocyte antigen-4; IL-8, interleukin-8; MDSCs, myeloid-derived suppressor cells PD-1, programmed cell death protein 1; PD-L1, programmed cell death ligand 1 TMB, tumor mutational burden.

Currently, the most studied biomarkers related to the efficacy of ICIs in TNBC are TILs, TMB, and PD-L1 expression status [**Table 1** (16, 18–38)]. PD-L1 as the target of anti-PD-L1 treatment is a potential predictive biomarker for the efficacy of PD-1/PD-L1 inhibitors and prognosis of TNBC (16, 19, 22, 39–41). However, the predictive value of PD-L1 is still questionable (23, 40, 42, 43). As mentioned above, TNBC patients have high levels of TILs and TMB. Previous studies have analyzed the predictive values of TILs and TMB for ICIs and have shown that they are associated with better efficacy in TNBC (24–26, 44–52). However, other studies have not confirmed the potential predictive value of TMB (53, 54). Myeloid-derived suppressor cells (MDSCs) and CTLA-4 are related to the increase of TNBC neoantigens, immunosuppression, and immune microenvironment; therefore, their value in IC inhibition cannot be ignored (55–59). Some studies have found that cytokines may predict the efficacy of ICIs and prognosis of BC, but there is a lack of consensus for TNBC. The following is an overview of the potential predictive and prognostic values (**Figure 1**), existing problems, and future application prospects of these biomarkers.

**Table 1:** Summary of clinical trials evaluating the predictive value of the biomarkers for ICIs in TNBC.

Biomarkers	Application	Trials	Treatment	N	Group	Key Data
**PD-L1**	Early TNBC	KEYNOTE-522 (29)	Pembro /placebo +chemotherapy	602	• PD-L1+	• pCR: 68.9% vs 54.9%
					PD-L1-	• pCR: 45.3% vs 30.3%
		Impassion031 (23)	Atezo /placebo +chemotherapy	333	• PD-L1+	• pCR: 69% (95% CI, 57-79) vs 49% (95% CI, 38-61)
					• PD-L1-	• pCR: 48% vs 34%
	Advanced TNBC	Impassion130 (16, 18)	Atezo /placebo + nab-paclitaxel	902	• PD-L1 +	• m PFS: 7.5 (95%CI, 6.7-9.2) mo vs 5.0 (95%CI, 3.8-5.6) mo; HR=0.64 (0.51-0.80)
						• m OS: 25.4 (95% CI, 19.6-30.7) mo vs 17.9 (95%CI, 13.6-20.3)mo; HR=0.67 (0.53-0.86)
					• PD-L1-	• m PFS: 5.6 mo vs 5.6 mo; HR=0.95 (0.79-1.15)
						• m OS: 19.7 mo vs 19.7 mo; HR=1.05 (0.87-1.28)
		KEYNOTE-012 (30)	single-agent pembro	111	• PD-L1+	• m PFS: 1.9 (95% CI, 1.7-5.5) mo
						• m OS: 11.2 (95% CI, 5.3- (not reached)) mo
		KEYNOTE-086 (31, 32)	single-agent pembro	Cohort A:170 B:84	• Cohort A (PD-L1+vs PD-L1-)	• m PFS: 2.0 (95%CI, 1.9-2.1) mo vs 1.9 (95%CI, 1.7-2.0) mo
						• m OS: 8.8 (95%CI, 7.1-11.2) mo vs 9.7 (95%CI, 6.2-12.6) mo
					• Cohort B (PD-L1+)	• m PFS: 2.1 (95%CI, 2.0-2.2) mo
						• m OS: 18.0 (95%CI, 12.9, 23.0) mo
**PD-L1**	Advanced TNBC	KEYNOTE-119 (20)	Pembro/ chemotherapy^i^	1098	• CPS ≥1	• m OS: 10.7 (95% CI, 9.3-12.5) mo vs 10.2 (95% CI, 7.9-12.6) mo; HR=0.86(0.69-1.06)
					• CPS ≥10	• m OS: 12.7(95% CI, 9.9-16.3) mo vs 11.6 (95% CI, 8.3-13.7) mo; HR=0.78(0.57-1.06)
					• CPS ≥20	• m OS: 14.9 mo vs 12.5 mo; HR=0.58(0.38-0.88)
		KEYNOTE-355 (19, 33)	Pembro /placebo+ chemotherapy	847	• CPS ≥1	• m PFS: 7.6 (95% CI, 6.6-8.0) mo vs 5.6 (95% CI, 5.4-7.4) mo; HR=0.75 (0.62-0.91)
						• m OS: 17.6(95% CI, 15.5-19.5) mo vs 16.0 (95% CI, 12.8-17.4) mo; HR=0.86 (0.72-1.04)
					• CPS ≥10	• m PFS: 9.7 (95% CI, 7.6-11.3) mo vs 5.6 (95% CI, 5.3-7.5) mo; HR=0.66 (0.50-0.88)
						• m OS: 23.0(95% CI, 19.0-26.3) mo vs 16.1 (95% CI, 12.6-18.8) mo; HR=0.73(0.55-0.95)
		JAVELIN (22)	single-agent avelumab	168 (58 was TNBC)	• TNBC (PD-L1+ vs PD-L1-)	• ORR: 22.2% vs. 2.6%
					• ≥1% TC (PD-L1+ vsPD-L1-)	• mPFS:5.9(95%CI, 5.7-6.0)weeks vs 6.0(95% CI, 5.9-6.0) weeks; HR=1.183 (0.815-1.716)
						• m OS: 6.5 (95% CI, 3.7-9.2) mo vs 8.3 (95% CI 6.3, ne) mo; HR=1.331 (0.815-2.174)
					• ≥5% TC (PD-L1+ vsPD-L1-)	• mPFS:6.0(95% CI, 5.7-7.1)weeks vs 5.9(95%CI, 5.9-6.0) weeks; HR=0.782 (0.473-1.290)
						• m OS: 6.5 (95% CI, 2.2-ne) mo vs 7.1 (95% CI, 5.1-11.3) mo; HR=1.057 (0.556-2.010)
					• ≥25% TC (PD-L1+ vsPD-L1-)	• mPFS:6.0(95% CI 5.4- ne)weeks vs 5.9(95% CI 5.9- 6.0) weeks; HR=0.695 (0.172-2.813)
						• m OS: 9.2 (95% CI, ne-ne) mo vs 6.8 (95% CI, 4.9-10.8) mo; HR=0.441 (0.061-3.177)
					• ≥10% IC c (PD-L1+ vsPD-L1-)	• mPFS:6.1(95%CI, 2.3-24,1)weeks vs 5.9(95%CI, 5.9-6.0)weeks; HR=0.656 (0.341-1.263)
						• m OS: 11.3 (95% CI, 1.4-ne) mo vs 6.8 (95% CI, 4.7-9.2) mo; HR=0.620 (0.250-1.541)
		KEYNOTE-150 (34)	Eribulin +pembro	107	• PD-L1+	• m PFS: 4.1 (95%CI, 2.1-4.8) mo
					• PD-L1-	• m PFS: 4.1 (95%CI, 2.3-6.3)mo
		Impassion131 (21)	Atezo/ placebo +paclitaxel	651	• PD-L1 +	• m PFS: 6.0 (95% CI 5.6-7.4) mo vs 5.7 (95% CI 5.4-7.2) mo; HR=0.82 (0.60-1.12)
						• Final OS: 22.1(95%CI 19.2-30.5) mo vs 28.3 (95% CI 19.1-NE) mo; HR=1.11(0.76-1.64)
**TILs**	Early TNBC	KEYNOTE-173 (35)	Pembro + chemotherapy	60	• Available pre-treatment sTILs date of ypT0/Tis ypN0	• pCR : 60% vs 40% ^a^
					• Available on-treatment sTILs date of ypT0/Tis ypN0	• pCR : 57% vs 43% ^b^
					• Available pre-treatment sTILs date of ypT0 /ypN0	• pCR: 58% vs 42% ^c^
					• Available on-treatment sTILs date of ypT0 /ypN0	• pCR: 53% vs 47%^d^
		GeparNuevo (28)	Durva / placebo+ chemotherapy	174	• Durvalumab-arm (sTILs)^e^	• OR: 1.23 (95%CI, 1.04-1.6)
					• Durvalumab-arm (iTILs)^e^	• OR: 1.58 (95%CI, 0.85-2.97)
					• Durvalumab-arm (iTILs post-pre)^f^	• OR: 5.15 (95%CI, 1.1-24.05)
					• Placebo-arm (sTILs) ^e^	• OR: 1.39 (95%CI, 1.12-1.74)
					• Placebo-arm (iTILs) ^e^	• OR: 0.94 (95%CI, 0.73-1.22)
					• Placebo-arm (iTILs post-pre)^f^	• OR: 1.19 (95%CI, 0.65-2.17)
	Advanced TNBC	KEYNOTE-086 (27, 31, 32)	single-agent pembro	^•^Cohort A: 147 B:46	• Cohort A	• ORR: 6% vs 2%^g^
					• Cohort B	• ORR: 39% vs 9%^h^
**TILs**	Advanced TNBC	Impassion130 (24, 36, 37)	Atezo/ placebo + nab-paclitaxel	902	• Any PD-L1, sTILs<10%	•m PFS: 5.6 mo vs 5.4 mo; HR=0.86 (0.73-1.02)
						•m OS: 19.2 mo vs 18.1 mo; HR=0.88 (0.72-1.08)
					• Any PD-L1, sTILs≥10%	•m PFS: 8.3 mo vs 6.1 mo; HR=0.64 (0.50-0.84)
						•m OS: 25.0 mo vs 20.0 mo; HR=0.75 (0.54-1.03)
					• PD-L1 ≥1%, sTILs<10%	•m PFS: 6.4 mo vs 4.7 mo; HR=0.80 (0.59-1.10)
						•m OS: 19.1 mo vs 17.6 mo; HR=0.74 (0.50-1.10)
					• PD-L1 ≥1%, sTILs≥10%	•m PFS: 9.0 mo vs 5.4 mo; HR=0.54 (0.39-0.75)
						•m OS: 30.0 mo vs 18.2 mo; HR=0.54 (0.39-0.75)
					• PD-L1 <1%, sTILs<10%	•m PFS: 5.6 mo vs 5.5 mo; HR=0.90 (0.73-1.10)
						•m OS: 19.3 mo vs 18.2 mo; HR=0.95 (0.75-1.20)
					• PD-L1 <1%, sTILs≥10%	•m PFS: 7.2 mo vs 9.0 mo; HR=0.92 (0.59-1.44)
						•m OS: 23.7 mo vs 24.5 mo; HR=1.04 (0.59-1.82)
					• Any PD-L1, CD8 <0.5%	•m PFS: 5.6 mo vs 5.6 mo; HR=0.86 (0.65 to 1.14)
						•m OS: 16.3 mo vs 22.3 mo; HR=1.16 (0.81 to 1.65)
					• Any PD-L1, CD8 ≥0.5%	•m PFS:7.4 mo vs 5.5 mo; HR=0.75 (0.62 to 0.91)
						•m OS: 22.6 mo vs 18.1 mo; HR=0.69 (95%CI, 0.54-0.81)
					• PD-L1 ≥1%,CD8<0.5%	•m PFS: 9.2 mo vs3.8 mo; HR=0.33 (0.13 to 0.83)
						•m OS:30.7 mo vs19.4 mo; HR=0.22 (0.06 to 0.90)
					• PD-L1 ≥1%,CD8 ≥0.5%	•m PFS: 7.7 mo vs 5.3 mo; HR=0.64 (0.49 to 0.83)
						•m OS: 28.6 mo vs 17.7 mo; HR=0.63 (0.46 to 0.86)
					• PD-L1<1%,CD8 <0.5%	•m PFS: 5.6 mo vs 5.7 mo; HR=1.00 (0.73 to 1.37)
						• m OS: 15.5 mo vs 22.3 mo; HR=1.39 (0.95 to 2.03)
					• PD-L1<1%,CD8 ≥0.5%	•m PFS: 6.5 mo vs 7.2 mo; HR=0.91 (0.68 to 1.21)
						•m OS: 21.0 mo vs 19.6 mo; HR=0.78 (0.56 to 1.10)
**TMB**	Early TNBC	GeparNuevo (26)	Durva / placebo+ chemotherapy	149^j^	• Durvalumab-arm (TMB≥2.05 muts/mb vs <2.05 muts/mb)	• pCR: 63% vs 40%^k^
					• Placebo-arm (TMB≥2.05 muts/mb vs <2.05 muts/mb)	•pCR: 52% vs 37%^l^
	Advanced TNBC	KEYNOTE-119 (25)	Pembro/ chemotherapy	253^i^	• TMB ≥10	•ORR: 14.3% (95%CI, 4.0-39.9) vs 8.3% (95%CI, 0.4-35.4)
					• TMB<10	•ORR: 12.7% (95%CI, 7.9-19.9) vs 12.8% (95%CI, 7.8-20.4)
**IL-8**	Advanced TNBC	A phase II trial (38, 129)	Camrelizumab +apatinib	28^m^	• Responder vs non-respondern	•Levels of IL-8: 0 pg/ml vs 2.15 pg/ml^O^

N, number of patients; TC, tumor cells; IC, immune Cells; m PFS, median PFS; mo, months; m OS, median OS; HR, hazard ratio, HR(95%CI); Pembro, Pembrolizumab; Atezo, Atezolizumab; OR, odds ratio; Durva, durvalumab.

^a^: Levels of TILs: Median (IQR): 42% (95% CI,10-74) vs 10% (95% CI,5-25); ^b^: Levels of TILs: Median (IQR): 65% (95% CI,5-89) vs 25% (95% CI,2-48); ^c^: Levels of TILs: Median (IQR): 40% (95% CI,10-75) vs 10% (95% CI,5-38); ^d^: Levels of TILs: Median (IQR): 65% (95% CI,5-86) vs 25% (95% CI,3-60); ^e^: pre-therapeutic; ^f^: difference of iTIL between post-window and pretherapeutic biopsy; ^g^: Levels of TILs: Median (IQR): 10% (95% CI,7.5-25) vs 5% (95% CI,1-10); ^h^: Levels of TILs: Median (IQR): 50% (95% CI,5-70) vs 15% (95% CI,5-37.5); ^i^: TMB data were available for 253/601 (42.1%) treated patients (pembro, n = 132; chemo, n = 121); ^j^: both whole exome sequencing and RNA-Seq data can be got from pretreatment samples of 149 TNBC of GeparNuevo; ^k^: P=0.028; ^l^: P=0.232; ^m^: 28 Patients had biopsies and blood collected; ^n^: responders (partial response ); non-responders (stable disease or progressive disease); ^o^: P = 0.001.

### 2.1 ICs

#### 2.1.1 PD-L1

PD-L1 is the ligand of PD-1 and is related to immunosuppression. Under normal circumstances, the immune system reacts to foreign antigens in the lymph nodes or spleen by promoting activation of antigen-specific cytotoxic T cells (such as CD8^+^ T cells). PD-1 combined with PD-L1 can transmit inhibitory signals and reduce the proliferation of CD8^+^ T cells in lymph nodes, which leads to immune escape of tumor cells. The PD-1/PD-L1 inhibitors interrupt binding of PD-L1 to PD-1, and in this way, tumor cells cannot transmit inhibitory signals to T cells, and T cells recognize and destroy cancer cells. About 20% of TNBC cells express PD-L1 (15, 60). Several studies have explored the predictive value of PD-L1 for immunotherapy in TNBC (16, 19, 22, 39–41). However, inconsistent results have been shown in different studies (23, 40, 42, 43).

Some studies have provided evidence about the predictive value of PD-L1 for efficacy of ICIs in TNBC [**Table 2** (18–21–23, 29–34, 37)]. PD-L1 has been shown to predict the efficacy of PD-1/PD-L1 inhibitors in mTNBC, whether in monotherapy or combination therapy (22, 39). Atezolizumab-treated patients with advanced TNBC in the PD-L1-positive population had a higher objective response rate (ORR) compared with the PD-L1-negative population (22.2% vs 2.6%) (39). Similarly, the JAVELIN study reported that mTNBC patients with higher PD-L1 expression had better efficacy of atezolizumab (22). The IMpassion 130 and KEYNOTE-355 studies indicated that PD-L1 positivity was related to better efficacy of PD-1/PD-L1 inhibitors in mTNBC (16, 17, 19). These studies have suggested that PD-L1 expression can identify patients who will benefit from ICIs.

**Table 2:** The predictive value of PD-L1 for PD-1/PD-L1 inhibitors in TNBC.

Application	Agents	Study	Combined Drug	N	Scoring Criteria	Group	Results
Early TNBC	Pembro	KEYNOTE-522 (29)	Pembro/placebo +chemotherapy^g^	602	CPS:1	•PD-L1+	• pCR: 68.9% vs 54.9%
						•PD-L1-	• pCR: 45.3% vs 30.3%
	Atezo	Impassion031 (23)	Atezo/placebo +chemotherapy^h^	333	PD-L1 in IC: 1%	• PD-L1 +	• pCR: 69% (95% CI, 57-79) vs 49% (95% CI, 38-61)
						• PD-L1 -	• pCR: 48% vs 34%
Advanced TNBC	Atezo	Impassion130 (18, 37)	Atezo/placebo + nab-paclitaxel	902	PD-L1 in IC: 1%	• PD-L1 +	• ORR: 58.9% (51.5-66.1) vs 42.6% (35.4-50.1) HR=1.96 (1.29-2.98)
							• m PFS: 7.5 (95%CI, 6.7-9.2) mo vs 5.0 (95%CI, 3.8-5.6) mo HR=0.64 (0.51-0.80)
							• m OS: 25.4 (95% CI, 19.6-30.7)mo vs 17.9 (95%CI, 13.6-20.3)mo HR=0.67 (0.53-0.86)
						• PD-L1 -	• m PFS: 5.6 mo vs 5.6 mo HR=0.95 (0.79-1.15)
							• m OS: 19.7 mo vs 19.7 mo HR=1.05 (0.87-1.28)
	Atezo	Impassion131 (21)	Atezo/ placebo +paclitaxel	651	PD-L1 in IC: 1%	• PD-L1 +	• m PFS: 6.0 (95% CI 5.6-7.4) mo vs 5.7 (95% CI 5.4-7.2) mo HR=0.82 (0.60-1.12)
							• Final OS: 22.1 (95% CI 19.2-30.5) mo vs 28.3 (95% CI 19.1-NE) mo HR=1.11(0.76-1.64)
Advanced TNBC	Pembro	KEYNOTE-012 (30)	single-agent pembro	111	PD-L1 in IC: 1%	• PD-L1+	• ORR: 18.5% (95% CI, 6.3-38.1)
							• m PFS: 1.9 (95% CI, 1.7-5.5) mo
							• m OS: 11.2 (95% CI, 5.3- (not reached)) mo
	Pembro	KEYNOTE-086 (31, 32)	single-agent pembro	Cohort A:170 B:84	CPS: 1	• Cohort A (PD-L1+)	• ORR: 5.7% (95%CI, 2.4-12.2)
							• m PFS: 2.0 (95%CI, 1.9-2.1) mo
							• m OS: 8.8 (95%CI, 7.1-11.2) mo
						• Cohort A (PD-L1-)	• ORR: 4.7% (95%CI, 1.1-13.4)
							• m PFS: 1.9 (95%CI, 1.7-2.0) mo
							• m OS: 9.7 (95%CI, 6.2-12.6) mo
						• Cohort B (PD-L1+)	• ORR: 21.4% (95%CI, 13.9-31.4)
							• m PFS: 2.1 (95%CI, 2.0-2.2) mo
							• m OS: 18.0 (95%CI, 12.9, 23.0) mo
	Pembro	KEYNOTE-150 (34)	Eribulin +pembro	107	CPS: 1	•PD-L1+	•ORR: 25.7% (95%, 12.9-40.8)
							•m PFS: 4.1 (95%CI, 2.1-4.8) mo
						•PD-L1-	•ORR: 25.0% (95%, 12.5-39.8)
							•m PFS: 4.1 (95%CI, 2.3-6.3)mo
Advanced TNBC	Pembro	KEYNOTE-119 (20)	Pembro/ chemotherapy^i^	1098	CPS: 1, 10, 20	•CPS ≥1	•ORR: 12.3% (95%CI, 8.1-17.6) vs9.4% (95%CI, 5.8-14.3)
							•m OS: 10.7 (95% CI, 9.3-12.5) mo vs 10.2 (95% CI, 7.9-12.6) mo HR=0.86(0.69-1.06)
						•CPS ≥10	•ORR: 17.7% (95%CI, 10.7-26.8) vs9.2% (95%CI, 4.3-16.7)
							•m OS: 12.7(95% CI, 9.9-16.3) mo vs 11.6 (95% CI, 8.3-13.7) mo HR=0.78(0.57-1.06)
						•CPS ≥20	•ORR: 26.0% vs12.0%
							•m OS: 14.9 mo vs 12.5 mo HR=0.58(0.38-0.88)
	Pembro	KEYNOTE-355 (19, 33)	Pembro/placebo + chemotherapy^j^	847	CPS: 1 and 10	•CPS ≥1	•ORR: 44.9% (95% CI, 40.1-49.8) vs 38.9% (95% CI, 32.2-45.8)
							•m PFS: 7.6 (95% CI, 6.6-8.0) mo vs 5.6 (95% CI, 5.4-7.4) mo HR=0.75 (0.62-0.91)
							•m OS: 17.6(95% CI, 15.5-19.5) mo vs 16.0 (95% CI, 12.8-17.4) mo HR=0.86 (0.72-1.04)
						•CPS ≥10	•ORR: 52.7% (95% CI, 45.9-59.5) vs 40.8% (95% CI, 31.2-50.9)
							•m PFS: 9.7 (95% CI, 7.6-11.3) mo vs 5.6 (95% CI, 5.3-7.5) mo HR=0.66 (0.50-0.88)
							•m OS: 23.0(95% CI, 19.0-26.3) mo vs 16.1 (95% CI, 12.6-18.8) mo HR=0.73(0.55-0.95)
Advanced Breast cancer	Avelumab	JAVELIN (22)	single-agent avelumab	168 (58 was TNBC)	•PD-L1 in TC^a^: 1, 5 and 25% •PD-L1 in IC^b^: 10%	• TNBC^c^ (PD-L1+ vs PD-L1-)	•ORR: 22.2% vs. 2.6%
						• ≥1% TC^d^ (PD-L1+ vsPD-L1-)	•ORR: 3.4% (95% CI, 0.3-8.2) vs 3.9% (95% CI, 0.5-13.5)
							•m PFS:5.9 (95% CI, 5.7-6.0) weeks vs 6.0 (95% CI, 5.9-6.0) weeks HR=1.183 (0.815-1.716)
							•m OS: 6.5 (95% CI, 3.7-9.2) mo vs 8.3 (95% CI 6.3, ne) mo HR=1.331 (0.815-2.174)
						• ≥5% TC^e^ (PD-L1+ vsPD-L1-)	•ORR: 4.3% (95% CI, 0.1, 21.9) vs 2.7% (95% CI, 0.6-7.6)
							•m PFS:6.0 (95% CI, 5.7-7.1) weeks vs 5.9 (95% CI, 5.9-6.0) weeks HR=0.782 (0.473-1.290)
							•m OS: 6.5 (95% CI, 2.2-ne) mo vs 7.1 (95% CI, 5.1-11.3) mo HR=1.057 (0.556-2.010)
						• ≥25% TC ^f^ (PD-L1+ vsPD-L1-)	•ORR: 0 (95% CI, 0-70.8) vs 3 (95% CI, 0.8-7.5)
							•m PFS:6.0 (95% CI 5.4- ne) weeks vs 5.9 (95% CI 5.9- 6.0) weeks HR=0.695 (0.172-2.813)
							•m OS: 9.2 (95% CI, ne-ne) mo vs 6.8 (95% CI, 4.9-10.8) mo HR=0.441 (0.061-3.177)
						• ≥10% IC ^c^ (PD-L1+ vsPD-L1-)	•ORR: 16.7 (95% CI, 2.1-48.4) vs 1.6 (95% CI, 0.2-5.7)
							•m PFS:6.1 (95% CI, 2.3-24,1) weeks vs 5.9 (95% CI, 5.9-6.0) weeks HR=0.656 (0.341-1.263)
							•m OS: 11.3 (95% CI, 1.4-ne) mo vs 6.8 (95% CI, 4.7-9.2) mo HR=0.620 (0.250-1.541)

N, number of patients; TC, tumor cells; IC, immune Cells; m PFS, median PFS; mo, months; m OS, median OS; HR, hazard ratio, HR (95%CI); Pembro, Pembrolizumab; Atezo, Atezolizumab.

^a^: the percentages of tumor cells expressing PD-L1: 1 and 5% thresholds with any staining intensity and a 25% threshold with moderate to high staining; ^b^: 10% of immune cells expressing PD-L1 at any staining intensity in tumor tissue; ^c^: ITT population, PD-L1+: PD-L1 expression≥10% immune cells; ^d^: ITT population, PD-L1+: PD-L1 expression≥1% tumor cells; ^e^: ITT population, PD-L1+: PD-L1 expression≥5% tumor cells; ^f^: ITT population, PD-L1+:PD-L1 expression≥25% tumor cells; ^g^: paclitaxel+carboplatin; ^h^: nab-paclitaxel + doxorubicin+ cyclophosphamide; ^i^: received investigator-choice (capecitabine, eribulin, gemcitabine, or vinorelbine); ^j^: nab-paclitaxel, paclitaxel, or gemcitabine-carboplatin.

Conversely, other clinical trials have not supported PD-L1 as a predictor of the efficacy of ICIs (23, 42). The KEYNOTE-522 and IMpassion 031 studies in early TNBC patients showed that, irrespective of positive or negative PD-L1 expression, PD-1/PD-L1 inhibitors combined with chemotherapy had a higher pathological complete remission (pCR) than placebo combined with chemotherapy (23, 42). Notably, the patients in these studies had early rather than advanced TNBC. The different results between early and advanced TNBC patients suggest that PD-is not an ideal biomarker and its predictive value varies according to individual immune function and/or disease setting. However, the potential mechanism underlying these results remains unclear.

The potential prognostic value of PD-L1 in TNBC remains contentious. Some studies have provided evidence that PD-L1-positive may be associated with better prognosis (40, 41, 61, 62). A meta−analysis reported that PD-L1-positive on tumor cells was related to poor prognosis, whereas PD-L1-positive on TILs was associated with better survival (41). Li et al. found that PD-L1 expression on TILs suggested better disease-free survival (DFS) in TNBC (61). However, Barrett et al. found that PD-L1-positive on tumor cells was associated with prolonged OS in patients with TNBC (40). Similarly, Botti et al. showed that PD-L1-positive on tumor cells was associated with better DFS in TNBC (62). However, other studies questioned the potential prognostic value of PD-L1 in TNBC (63, 64). A meta-analysis exploring the relationship between PD-L1 and prognosis in TNBC found no significant association between PD-L1 expression and OS (64). Thus, the prognostic value of PD-L1 in TNBC remains unclear and further studies are required.

The inconsistency of these studies suggests that the PD-L1 expression is affected by factors such as complex immune environment and different detection methods. First, expression of PD-L1 is regulated by various mechanisms including the signal transducer and activator of transcription 3 and nuclear factor-κB pathways (65). Additionally, the function of PD-L1 is influenced by ubiquitination, glycosylation, phosphorylation and methylation (65). Therefore, expression of PD-L1 may be altered over time and be induced by other therapies. Second, the different antibodies or detection methods may have affected the results of PD-L1 expression in many studies (66–68). Antibodies for the detection of PD-L1, such as 28-8, 22C3, SP263 and SP142, have been approved as companion/complementary diagnostics to nivolumab, pembrolizumab, durvalumab and atezolizumab, respectively (69). In clinical application, the major differences among the four antibodies are mainly stained cells and scoring criteria of PD-L1. The Blueprint Project showed that 28-8, 22C3 and SP263 mainly stained tumor cells, and the test results were similar. SP142 stained immune cells more prominently than the other antibodies did. Compared with tumor cell staining, immune cell staining was more heterogeneous (70, 71). At present, there are four scoring criteria of PD-L1: combined positive score (CPS), tumor proportion score, immune cell score and tumor cell score (72, 73). Because of the different scoring criteria, the definitions of PD-L1-positive tumors were different. For example, PD-L1-positive tumors in the IMpassion 130 study were defined as staining of any intensity in immune cells occupying ≥1% of the tumor area tested by SP142 (16). In the KEYNOTE-355 study, PD-L1-positive tumors were defined as CPS ≥1 and CPS ≥10, where CPS was the ratio of PD-L1-positive cells (tumor cells, lymphocytes and macrophages) to the total number of tumor cells tested by 22C3, multiplied by 100 (19). However, these four evaluation methods have not been comprehensively compared; therefore, which method can better reflect the expression level and predictive value of PD-L1 requires further study. Additionally, there were temporal and spatial differences in PD-L1 expression between primary and metastatic lesions (74). Expression of PD-L1 in the metastatic site was significantly lower compared with the primary site (75, 76). Factors such as the empirical judgment of pathologists, heterogeneity of PD-L1, and the effect of drugs might also interfere with PD-L1 expression (65, 70, 77–79).

In summary, the potential predictive and prognostic values of PD-L1 in TNBC remain controversial. Understanding of the tumor, microenvironment, and host factors that influence response to ICIs may contribute to identifying more reliable biomarkers (80). Accurate methods are needed to detect PD-L1 expression and guide precision medicine (81). At present, identifying patients who can benefit from ICIs partly relies on immunohistochemical assays used in clinical trials (82). However, it is difficult to detect the dynamic change in PD-L1 expression, and some factors can interfere with the results. Therefore, the determination of the optimal assay will require further rigorous studies. Scoring systems and thresholds for PD-L1 positivity lack standardization, and this may affect the judgment of PD-L1 positivity. Fortunately, this study area is rapidly developing and PD-L1 as a potential prognostic and predictive biomarker will be fully optimized for TNBC in the future.

#### 2.1.2 CTLA-4

CTLA-4 is one of the immunoglobulin superfamily and a signal receptor on the T-cell membrane (83). It is homologous to CD28 on the surface of T cells and competes with CD28 to bind to B7-1 (CD80) and B7-2 (CD86) on antigen-presenting cells, although it has a stronger affinity for B7-1 and B7-2 (84). When B7 binds to CD28, it initiates an activation signal, which is inhibited when CTLA-4 binds to B7 (85). Normally, CTLA-4 participates in negative immunoregulation. However, tumors can also participate in these immunoregulatory pathways by expressing CTLA-4, which decreases immune cell functions (86, 87). Some studies have found that the high levels of CTLA-4 correlate with better efficacy of anti-CTLA-4 therapies in melanoma (88, 89). However, there is a lack of data from clinical trials about its predictive value for ICIs in TNBC. In addition to the above, the potential prognostic value of CTLA-4 in BC has been reported (55, 56). Yu et al. analyzed tissue samples from 130 BC patients who underwent surgery. They found that more interstitial CTLA-4^+^ lymphocytes were related to longer DFS and OS, whereas more CTLA-4^+^ tumor cells were related to shorter DFS and OS (55). Lu et al. analyzed an RNA-sequencing dataset and found that BC patients with high CTLA-4 expression had a significantly elevated risk of death compared with those with low CTLA-4 expression (56).

Thus, CTLA-4 expression in BC may be a potential prognostic biomarker. However, whether these results can be applied to TNBC is worthy of further investigation. The potential predictive value of CTLA-4 for efficacy of ICIs in TNBC has not been clarified. Relevant research should be carried out in the future to explore the potential prognostic and predictive value of CTLA-4 in TNBC.

### 2.2 Immune Cells

#### 2.2.1 TILs

TILs are heterogeneous lymphocyte groups that exist in tumor nests and interstitial cells. They are dominated by different degrees of monocyte and lymphocyte infiltration. The percentage of TILs is higher in TNBC than in luminal type and HER2-enriched BC (46, 90). Some studies have reported that the quantity of TILs in TNBC has predictive value for efficacy of ICIs [**Table 3** (27, 31, 32, 35)]. In the KEYNOTE-086 study, Sherene et al. found that high ORR for mTNBC patients treated with pembrolizumab was associated with high level of TILs (27). Similar findings were reported in the KEYNOTE-173 study, where a high level of TILs was significantly related to better pCR or ORR for TNBC patients treated with pembrolizumab (44). Loi et al. found that stromal TILs ≥5% predicted the response to pembrolizumab monotherapy (45). The biomarker analyses of the GeparNuevo trial showed that higher level of stromal TILs was associated with pCR in the overall cohort but did not predict the efficacy of durvalumab (28). The increased level of intratumoral TILs from before to after treatment was predictive for pCR specifically in the durvalumab arm (28). An increase in TILs in early TNBC patients after neoadjuvant therapy was associated with improved DFS and OS (46, 47). A phase III trial reported an approximately 15% reduction in death and recurrence for every 10% increase in TILs (47). At present, several studies have demonstrated the predictive value of TILs, but there is a lack of high-quality evidence. Therefore, the predictive value of TILs for efficacy of ICIs in TNBC remains contentious.

**Table 3:** Results of the exploratory studies of TILs in TNBC patients treated with PD-1/PD-L1 inhibitors.

Application	Agents	Study	Combined Drug	N	Group	Results	Levels of TILs
Early TNBC	Pembro	KEYNOTE-173 (35)	Pembro + chemotherapy^a^	60^b^	•Available pre-treatment sTILs date of ypT0/Tis ypN0	•pCR :60% vs 40% ^h^	•Median (IQR): 42% (95% CI,10-74) vs 10% (95% CI,5-25) ^c^ $
					•Available on-treatment sTILs date of ypT0/Tis ypN0	•pCR :57% vs 43% ^h^	•Median (IQR): 65% (95% CI,5-89) vs 25% (95% CI,2-48) ^e^ $
					•Available pre-treatment sTILs date of ypT0 /ypN0	•pCR :58% vs 42% ^h^	•Median (IQR): 40% (95% CI,10-75) vs 10% (95% CI,5-38) ^d^ $
					•Available on-treatment sTILs date of ypT0 /ypN0	•pCR :53% vs 47% ^h^	•Median (IQR): 65% (95% CI,5-86) vs 25% (95% CI,3-60) ^f^ $
Advanced TNBC	Pembro	KEYNOTE-086 (27, 31, 32)	single-agent pembro	^•^Cohort ^i^ A: 147 B:46	•Cohort A	•ORR :6% vs 2%^j^	•Median (IQR): 10% (95% CI,7.5-25) vs 5% (95% CI,1-10) ^k^ $
					•Cohort B	•ORR :39% vs 9%^j^	•Median (IQR): 50% (95% CI,5-70) vs 15% (95% CI,5-37.5) ^k^ $
Advanced TNBC	Atezo	Impassion130 (24, 36, 37)	Atezo/ placebo + nab-paclitaxel	902	•Any PD-L1, sTILs<10%	•m PFS: 5.6 mo vs 5.4 mo^l^ HR=0.86 (0.73-1.02)	•sTILs<10%, any CD8
						•m OS: 19.2 mo vs 18.1 mo^l^ HR=0.88 (0.72-1.08)	
					•Any PD-L1, sTILs≥10%	•m PFS: 8.3 mo vs 6.1 mo^l^ HR=0.64 (0.50-0.84) $	•sTILs≥10%, any CD8
						•m OS: 25.0 mo vs 20.0 mo^l^ HR=0.75 (0.54-1.03)	
					PD-L1 ≥1%, sTILs<10%	•m PFS: 6.4 mo vs 4.7 mo^l^ HR=0.80 (0.59-1.10)	•sTILs<10%, any CD8
						•m OS: 19.1 mo vs 17.6 mo •HR=0.74 (0.50-1.10)	
					PD-L1 ≥1%, sTILs≥10%	•m PFS: 9.0 mo vs 5.4 mo^l^ HR=0.54 (0.39-0.75) $	•sTILs≥10%, any CD8
						•m OS: 30.0 mo vs 18.2 mo^l^ •HR=0.54 (0.39-0.75) $	
					PD-L1 <1%, sTILs<10%	•m PFS: 5.6 mo vs 5.5 mo^l^ HR=0.90 (0.73-1.10)	•sTILs<10%, any CD8
						•m OS: 19.3 mo vs 18.2 mo^l^ HR=0.95 (0.75-1.20)	
					PD-L1 <1%, sTILs≥10%	•m PFS: 7.2 mo vs 9.0 mo^l^ HR=0.92 (0.59-1.44)	•sTILs≥10%, any CD8
						•m OS: 23.7 mo vs 24.5 mo^l^ HR=1.04 (0.59-1.82)	

N, number of patients; TC, tumor cells; IC, immune Cells; IQR, interquartile range; $, indicates statistical significance; pCR, pathological complete response.

^a^: Pembro + taxane with or without carboplatin, and then doxorubicin and cyclophosphamide before surgery; ^b^: 53 patients have pre-treatment sTILs data and 49 patients have on-treatment sTILs data; ^c^: Median (IQR) TIL level in responders vs non-responders, P= 0.0059, AUROC (90% CI) 0.653 (0.527-0.779); ^d^: Median (IQR) TIL level in responders vs non-responders, P= 0.0091, AUROC (90% CI) 0.638 (0.512-0.764); ^e^: Median (IQR) TIL level in responders vs non-responders, P= 0.0085, AUROC (90% CI) 0.690 (0.564-0.817); ^f^: Median (IQR) TIL level in responders vs non-responders, P= 0.0097, AUROC (90% CI) 0.676 (0.547-0.806); ^g^: DCR (CR + PR + SD ≥ 24 weeks; ^h^: Number of responders/number vs Number of no-responders/number, and patients not assessable for pCR were considered non-responders; ^i^: 193 patients had evaluable tumor samples: 147 from cohort A, 46 from cohort B; ^j^: ORR in patients with TIL level ≥vs<median; ^k^: Median (IQR) TIL level in responders vs non-responders, and patients without response data were counted as non-responders. Response data included complete response or partial response; ^l^: Atezo + nab-paclitaxel vs placebo + nab-paclitaxel.

Most of the above studies have focused on the predictive value of the level of TILs rather than TIL subsets for efficacy of ICIs in TNBC (27, 44–47). TIL subsets with different immune cell compositions represent different immune responses and prognosis (48, 91). On the one hand, TIL subsets can predict the efficacy of ICIs for TNBC. For example, mTNBC patients who received atezolizumab as monotherapy with intratumoral CD8^+^ T cells >1.35% prior to treatment presented trends toward higher ORR and longer OS (92). An exploratory analysis of the IMpassion 130 study reported that the percentage of CD8^+^ T cells (≥0.5%) was predictive for the efficacy of atezolizumab plus nab-paclitaxel in mTNBC (24). Similarly, Jiang et al. found that a high CD8 immunohistochemical score was associated with better efficacy of immunotherapy in the IM subtype of TNBC (93, 94). On the other hand, TIL subsets might be associated with worse prognosis in BC (50, 95). For example, a high enrichment score of immature DCs and eosinophils is associated with poor OS (95). Additionally, lymphocytes with positive expression of fork head box protein 3 in tumor tissues are significantly associated with poor prognosis in BC (50).

Taken together, the potential predictive value of TILs in TNBC needs further exploration, and TILs may have potential prognostic value in TNBC. The 17th St Gallen International Breast Cancer Consensus Conference has shown that TILs may serve as a prognostic biomarker in TNBC (96). The following factors may affect the predictive effect of TILs. First, the evaluation of TILs is still mainly dependent on pathologists, who may obtain different results. Because the composition of TILs is complex, it is a challenge for researchers to distinguish the functions of different cells and their predictive values. Restrictions among TILs and the influence of cytokines on their functions can also influence their predictive value. Fortunately, testing standards have been formed for TILs, and artificial intelligence has gradually been applied to case interpretation (97, 98). Therefore, TILs can be detected more objectively. In the future, the clinical application of TILs will have broad prospects in TNBC, but currently, we should not use TILs to select individual patients for ICIs in clinical practice.

#### 2.2.2 MDSCs

Myelopoiesis is a tightly regulated process that is altered in cancer, leading to the expansion of immature myeloid cells, now called MDSCs (99). Tumor cells secrete interleukin (IL)-8 to recruit MDSCs into the tumor microenvironment; inhibit T-cell activation by consuming and limiting cysteine and other essential amino acids, such as cysteine, for T-cell activation; induce immunosuppression; and promote tumor progression (100–104). MDSCs can be divided into two major groups: polymorphonuclear and monocytic MDSCs (105). Some studies have shown that the subsets of MDSCs are associated with the efficacy of ICIs in NSCLC and melanoma (106–108). However, there is no evidence whether the MDSCs are related to the efficacy of ICIs in TNBC. Therefore, the predictive value of MDSCs for efficacy of ICIs in TNBC is not clear.

Other studies have shown that higher levels of MDSCs are associated with worse prognosis in patients with solid tumors such as advanced BC (57, 58, 109, 110). Furthermore, advanced BC patients with circulating MDSCs >3.17% at baseline had poorer median OS than patients with circulating MDSCs ≤3.17% (5.5 vs 19.32 months) (57). In support of the prognostic value of MDSCs, Bergenfelz et al. observed 54 patients with metastatic BC and found that higher MDSC count was associated with worse PFS and OS (58).

As mentioned above, MDSCs may have potential prognostic value in BC, although no similar study has focused on TNBC. The potential predictive value of MDSCs for efficacy of ICIs in BC has not yet been clarified. Some studies have reported that ICIs reduce the number of circulating MDSCs, which implies that ICIs might have an MDSC-inhibiting effect (111, 112). Therefore, the detection of circulating MDSCs may contribute to a better understanding of the predictive value of MDSCs for efficacy of ICIs in TNBC. In the future, MDSCs are worth further exploration, especially for the potential predictive value of ICIs and prognostic value in TNBC.

### 2.3 TMB

Tumor formation and progression are accompanied by the acquisition and accumulation of mutations. TMB refers to the total number of base substitutions, somatic gene coding errors, and gene deletion or insertion errors detected per million bases (113). Exogenous DNA damage and DNA repair pathway defects can cause mutations. These mutations might lead to new antigens that are identified as foreign by the immune system, leading to activation of the immune microenvironment (114). Correspondingly, an activated immune microenvironment is favorable for tumor shrinkage by PD-1/PD-L1 inhibitors (115). In the Chinese population, the rate of TMB-high (TMB-H) in BC is higher than that reported by The Cancer Genome Atlas (116). Among the various subtypes of BC, TNBC has the highest TMB, followed by HER2-positive BC (117–119). Some trials reported that TMB-H was related to the better efficacy of immunotherapy in TNBC (25, 26, 51, 52). The KEYNOTE-119 study reported that ORR was significantly increased by single-agent pembrolizumab in mTNBC patients with TMB ≥10 mutations/Mb, while no significant difference was demonstrated in the ORR between chemotherapy and pembrolizumab in patients with TMB <10 mutations/Mb (25). The results of genome sequencing and whole exome sequencing from 3,369 BC patients also showed that patients with TMB ≥10 mutations/Mb might benefit from ICI treatment (51). Karn et al. performed whole exome sequencing in patients with early TNBC and obtained RNA data from pretreatment samples of patients treated with neoadjuvant ICIs (26). They found that TMB-H was associated with the efficacy of ICIs, and the pCR of patients with TMB-H and TMB-low in the durvalumab treatment arm was 63% and 40%, respectively (26). Barroso et al. analyzed 62 mTNBC patients who had previously been treated with ICIs alone or combined with another therapy (52). They found that TMB-H was associated with longer PFS among patients with mTNBC treated with anti-PD-1/PD-L1 therapies.

However, the predictive value of TMB in BC was questioned by other studies (53, 54). An analysis of 10,000 cases showed that patients with TMB-H BC treated with ICIs had worse efficacy than those who received other antitumor treatments (53). Additionally, Adams et al. found no relationship between PFS and TMB in patients with metastatic BC with TMB-H treated with pembrolizumab monotherapy (54). These results suggest that TMB-H may not have predictive value for efficacy of ICIs in BC. However, these trials did not report the BC subtypes, and whether these conclusions can be applied to TNBC requires further study.

The potential predictive value of TMB for efficacy of ICIs and its potential prognostic value in TNBC are unclear. TMB-H (≥10 mutations/Mb) is useful in certain circumstances to help define which BC patients can appropriately receive pembrolizumab, based on version 1.2021 of the NCCN guidelines for BC. However, there are still some unresolved issues for TMB in TNBC. First, the cutoff point of TMB-H is still uncertain and it has differed among trials. Even if the US Food and Drug Administration defines TMB-H as TMB ≥10 mutations/Mb, this definition is still controversial (120). Therefore, one of the challenges for the future application of TMB is to standardize the cutoff point of TMB. Not all TMB-H patients were positively correlated with a good therapeutic effect of ICIs. In some cases, tumor cells develop drug resistance because of TMB-H (121). For instance, as one of the forms of TMB, the deletion mutation of PTEN can promote tumor resistance to ICIs (122, 123). Therefore, clarifying correlations between mutation type and efficacy of ICIs in TNBC is important.

### 2.4 Cytokines

Cytokines are a class of soluble low-molecular weight proteins secreted by immune and nonimmune cells, including interleukins, tumor necrosis factors, interferons, colony-stimulating factors and transforming growth factors (124). Through the autocrine and paracrine pathways, cytokines can regulate proliferation, differentiation and function of immune cells, tumor microenvironment, and even affect the migration of cancer cells (124, 125). Recent studies have explored the relationship between cytokines and the efficacy of immunotherapy and prognosis in tumors (125, 126). Cytokines may be related to the efficacy of ICIs in solid tumors, such as NSCLC and melanoma (127–131). Schalper et al. found that patients with melanoma or NSCLC with high IL-8 levels derived limited benefit from nivolumab and/or ipilimumab (100). Patients with TNBC with low plasma IL-8 levels are more likely to respond to camrelizumab combined with apatinib (129). However, there is insufficient evidence to support the predictive value of IL-8 levels for efficacy of ICIs in TNBC. Other studies have suggested that IL-8 may have prognostic value in TNBC (132–134). Deng et al. found that IL-8 induced TNBC cell migration and tumor growth by multiple signaling pathways (132). Through bioinformatic analysis, Kim et al. and Malone et al. found that high IL-8 expression was associated with poor prognosis compared with low IL-8 expression in TNBC (133, 134).

In summary, there is a lack of consensus whether cytokines can be used to evaluate the efficacy of ICIs and prognosis in TNBC. Because of the complexity of the tumor microenvironment and interaction among cytokines, further exploration of cytokines may be difficult. Compared with invasive examinations such as needle biopsy, cytokines provide another noninvasive examination that can be dynamically detected. At present, cytokine therapy is important for some cancers and has achieved good clinical efficacy in melanoma (135), prostate cancer (136) and colorectal cancer (137). In the future, how to expand the clinical application of cytokines in TNBC is still a challenge.

## 3 Conclusions and Future Perspectives

ICIs are a promising treatment approach for TNBC. Several clinical trials have shown that ICIs improve the treatment outcomes of TNBC patients (16, 19, 22, 39–41). However, some patients do not respond to ICIs and may suffer immune-related adverse events. Therefore, it is important to evaluate biomarkers in TNBC to identify patients that might benefit from immunotherapy. In this review, we discussed different biomarkers related to the efficacy of ICIs and their potential prognostic value in TNBC, including TILs, PD-L1, cytokines and TMB. Among them, PD-L1 and TMB-H are regarded as criteria for screening BC patients who are suitable for pembrolizumab according to versions 1.2020 and 1.2021 of the NCCN guidelines for BC.

Although many studies of biomarkers for ICIs are underway, there are still some unresolved issues. First, some trials collect samples at a single time point, which lack basic information regarding the dynamic responses to ICIs. This can be overcome by collecting longitudinal tumor samples. Compared with the collection of tumor tissues, peripheral blood testing has the advantages of easy sample collection and causing little harm to patients. Therefore, liquid biopsy may have promise in clinical translational studies. Second, there is no unified detection method or standard for biomarkers such as PD-L1 or TILs. Different studies may obtain different conclusions when using the same biomarker. Third, new immunotherapeutic combinations are gradually emerging, and whether these predictive biomarkers are suitable for new regimens needs to be explored further. Fourth, some patients develop drug resistance in the course of receiving ICIs. Therefore, studies of biomarkers should not only focus on the prognosis and efficacy for ICIs in TNBC, but also the role of biomarkers in the mechanisms related to drug resistance. Finally, a single factor cannot accurately predict the prognosis and efficacy of ICIs in TNBC. In the future, the predictive value of composite biomarkers should be further explored.

In summary, many biomarkers are emerging as potential predictive markers for ICIs and prognostic biomarkers in TNBC, which still need further validation. New detection methods, such as high-throughput sequencing (138), single-cell sequencing technology (139) and magnetic resonance imaging computer-aided detection (a technology used to identify the TILs level) (140), are being applied to biomarker research. These methods will help identify new biomarkers and facilitate more convenient and accurate use of them in the clinic.

First, TMB might lead to new antigens and enhance immunogenicity. Second, the PD-1 combined with PD-L1 can transmit inhibitory signals and reduce immune activation, which leads to the immune escape of tumor cells. Third, CTLA-4 can compete with CD28 to bind to CD80 and CD86 on antigen-presenting cells (APC), and inhibit the activation signal. Fourthly, cytokines can regulate proliferation, differentiation and function of immune cells, tumor microenvironment, and even affect migration of cancer cells. Especially, tumor cells secrete IL-8 to recruit MDSCs into the tumor microenvironment to induce immunosuppression, and promote tumor progression. CTLA-4, cytotoxic T lymphocyte antigen-4; IL-8, interleukin-8; MDSCs, myeloid-derived suppressor cells PD-1, programmed cell death protein 1; PD-L1, programmed cell death ligand 1 TMB, tumor mutational burden.

## Author Contributions

Conceptualization: HL. Article collection and analysis: SY, DZ, YC, and XM. Manuscript writing: QT and SY. All authors contributed to the article and approved the submitted version.

## Funding

We would like to acknowledge the funding support of National Natural Science Foundation of China (Grant No. 81902713), Breast Disease Research Fund of Shandong Provincial Medical Association (Grant No. YXH2020ZX066) and Chinese Society of Clinical Oncology-Heng Rui Cancer Research Fund (Grant No. Y-HR2019-0432; Y-HR2018-121).

## Conflict of Interest

The authors declare that the research was conducted in the absence of any commercial or financial relationships that could be construed as a potential conflict of interest.

## Publisher’s Note

All claims expressed in this article are solely those of the authors and do not necessarily represent those of their affiliated organizations, or those of the publisher, the editors and the reviewers. Any product that may be evaluated in this article, or claim that may be made by its manufacturer, is not guaranteed or endorsed by the publisher.
